# Patient characteristics of, and remedial interventions for, complaints and medico-legal claims against doctors: a rapid review of the literature

**DOI:** 10.1186/s13643-024-02501-8

**Published:** 2024-04-09

**Authors:** Timothy J. Schultz, Michael Zhou, Jodi Gray, Jackie Roseleur, Richard Clark, Dylan A. Mordaunt, Peter D. Hibbert, Georgie Haysom, Michael Wright

**Affiliations:** 1 College of Medicine and Public Health, Flinders Health and Medical Research Institute, Flinders University, Adelaide, Australia; 2 College of Nursing and Health Sciences, Flinders University, Adelaide, Australia; 3 HealthFX, Melbourne, Australia; 4 Southern Adelaide Local Health Network, Adelaide, Australia; 5 Australian Institute of Health Innovation, Macquarie University, Sydney, Australia; 6 IIMPACT in Health, Allied Health and Human Performance, University of South Australia, Adelaide, Australia; 7 Avant Mutual, Sydney, Australia; 8 Centre for Health Economics Research and Evaluation, University of Technology Sydney, Sydney, Australia

**Keywords:** Complaints, Medico-legal claims, Communication and resolution program, Risk management program, Patient characteristics, Patient safety

## Abstract

**Background:**

It is uncertain if patient’s characteristics are associated with complaints and claims against doctors. Additionally, evidence for the effectiveness of remedial interventions on rates of complaints and claims against doctors has not been synthesised.

**Methods:**

We conducted a rapid review of recent literature to answer: Question 1 “What are the common characteristics and circumstances of patients who are most likely to complain or bring a claim about the care they have received from a doctor?” and Question 2 “What initiatives or interventions have been shown to be effective at reducing complaints and claims about the care patients have received from a doctor?”. We used a systematic search (most recently in July 2023) of PubMed, Scopus, Web of Science and grey literature. Studies were screened against inclusion criteria and critically appraised in duplicate using standard tools. Results were summarised using narrative synthesis.

**Results:**

From 8079 search results, we reviewed the full text of 250 studies. We included 25 studies: seven for Question 1 (6 comparative studies with controls and one systematic review) and 18 studies for Question 2 (14 uncontrolled pre-post studies, 2 comparative studies with controls and 2 systematic reviews). Most studies were set in hospitals across a mix of medical specialties.

Other than for patients with mental health conditions (two studies), no other patient characteristics demonstrated either a strong or consistent effect on the rate of complaints or claims against their treating doctors.

Risk management programs (6 studies), and communication and resolution programs (5 studies) were the most studied of 6 intervention types. Evidence for reducing complaints and medico-legal claims, costs or premiums and more timely management was apparent for both types of programs. Only 1 to 3 studies were included for peer programs, medical remediation, shared decision-making, simulation training and continuing professional development, with few generalisable results.

**Conclusion:**

Few patient characteristics can be reliably related to the likelihood of medico-legal complaints or claims. There is some evidence that interventions can reduce the number and costs of claims, the number of complaints, and the timeliness of claims. However, across both questions, the strength of the evidence is very weak and is based on only a few studies or study designs that are highly prone to bias.

**Supplementary Information:**

The online version contains supplementary material available at 10.1186/s13643-024-02501-8.

## Background

Up to 10% of hospital patients experience an adverse event [1]. Medical negligence or the failure to meet the standard of care reasonably expected of an ‘average’ doctor is a contributing factor to a small proportion of adverse events [1, 2]. Medico-legal claims seeking compensation for medical negligence may be filed against doctors by patients through civil litigation. For less serious events or to express dissatisfaction with care, patients may also make a formal complaint, either directly to their care provider or the provider’s employer or to medical and other regulators and health complaints entities [3].

Doctors’ demographic (e.g. gender, age, years spent in practice) and workplace-related factors (e.g. greater number of patient lists) are associated with the risk of complaints and malpractice claims [4, 5]. It is less clear what, if any, patient characteristics are associated with complaints and claims, and anecdotal evidence suggests that the rate of complaints and claims is rising [6]. Though females may be more likely to complain, and complaints and claims are often raised by patients’ living or bereaved relatives [7, 8], there are no relevant systematic reviews on this topic. This led to the following review question (Question 1) “What are the common characteristics and circumstances of patients who are most likely to complain or bring a claim about the care they have received from a doctor?”.

In addition to the impact on patient wellbeing, doctors involved in adverse events experience serious emotional and psychological impacts [9]. Additionally, the financial cost to health systems from medico-legal claims is significant, potentially jeopardising the long-term financial sustainability of some public health systems [10]. Doctors, hospitals, health services, health regulators, representative medical organisations and medical insurers are therefore all highly motivated to provide safe, high-quality care that minimises complaints and claims against them, their staff, stakeholders and members. For example, medical colleges, practitioner regulation boards and medical indemnity insurers maintain professional standards of their members and conduct activities such as continuing professional development (CPD) [11], remediation programs [12] and communication and resolution programs (CRPs) [13]. Despite a recent scoping review describing how remediation programs are delivered to regulated health professionals [14], there is no substantive review of the literature across the wide range of stakeholders and potential interventions applicable to reduce complaints and claims against doctors. We therefore posed the following additional review question (Question 2): “What initiatives or interventions have been shown to be effective at reducing complaints and claims about the care patients have received from a doctor?” [6].

### Review objective and research questions

The purpose of this review was to provide an evidence-based foundation to understand which patient factors influence complaints or claims and what interventions can support a reduction in complaints or claims [6]. This information could be used by clinicians, hospital administrators, healthcare regulators and medical indemnity insurers to inform their practice and policy. For the purposes of this study, a “claim” was defined as an assertion of wrongdoing that forms the basis for a request for compensation [15]; an “unwarranted” claim occurred when the care provided had not been below the expected standard and the complaint was not otherwise warranted [6].

## Methods

A protocol defining the scope of the review (PEO/PICO, inclusion and exclusion criteria, search strategy and limits) was developed according to Sax Institute guidelines [16] but was not prospectively registered. The review was conducted according to guidance provided by the Cochrane Rapid Review method [17] and the SelecTing Approaches for Rapid Reviews (STARR) approach [18]. The updated Preferred Reporting Items for Systematic Reviews and Meta-Analysis (PRISMA) checklist was used to report review findings [19].

### Scope of the review

The review focussed on health systems of high-income Commonwealth countries including Australia, New Zealand, Canada and the United Kingdom (UK). Additionally, studies from the United States of Amercia (USA), Ireland and Western Europe were included to inform the review. The review focussed on the peer-reviewed literature although grey literature of similar quality was also searched. The review was conducted over an 8-week period from September to October 2022. The search was repeated in September 2023.

### Inclusion and exclusion criteria

The inclusion and exclusion criteria for Question 1 and Question 2 are included in Table 1. The settings were hospitals (excluding the emergency department), primary care and secondary care. Regulatory complaints, complaints to practices or hospitals and claims for compensation were included, while complaints on social media were excluded. For Question 1, the review focussed on correlations between the ‘exposure’ (e.g. patient characteristics) and the number, type or nature of complaints/claims. For Question 2, the review included interventions implemented primarily to reduce the number of complaints/claims against doctors, although other secondary outcomes included the costs of claims or insurance premiums, the duration of the claims management process, doctor risk profile or performance, doctor confidence/knowledge/satisfaction, workplace culture, and patient outcomes (e.g. morbidity) or patient satisfaction.

**Table 1:** Summary of inclusion and exclusion criteria for the two review questions (RQs)

	Inclusion criteria (both Question 1 and Question 2)	
Setting	Inpatient, outpatient, primary and secondary care; public & private; high income countries; English language	
Care type	Chronic care, acute care, surgical and hospital interventions	
Complaints/claims	Regulatory or direct to practice/hospital complaints. Litigated or unlitigated claims	
Study design	Systematic reviews, randomised controlled trials, cohort, case–control, interrupted time series, pre-post	
	**Question 1—patient characteristics**	**Question 2—remedial interventions**
Participants	Patients and family members	Doctors
Exposure/intervention characteristics	Patient socio-demographics (e.g. age, gender, nationality), diagnosis, medical history, relationship with doctor, setting, family involvement	Education for doctors including communication and risk mitigation strategies, workflow, change roles and responsibilities
Outcomes	Number or rate of complaints/claims	Number or rate of complaints/claims, claims management, patient or doctor satisfaction, doctor risk profile or performance, doctor confidence

Only English language studies using quantitative study designs included in the National Health and Medical Research Council (NHRMC) guidelines [20] were included (e.g. ranging from level I systematic review, level II randomised controlled trial, level III pseudorandomised trial/comparative study with or without concurrent controls, and level IV case series with either post-test or pre-test/post-test outcomes). Cross-sectional studies were excluded.

### Search strategy and selection criteria

Given the aetiological nature of studies relevant to Question 1 in particular, we used a PEO approach (Participant, Exposure, Outcome) [21] to frame the search strategy (see Supplementary Table S1, S2, S3). Terms relating to ‘participants’ included doctors and health services. Terms relating to ‘exposure’ included patient characteristics (such as demographics, socio-economic status, and health literacy) for Question 1, and patient safety interventions (such as checklists, care bundles and teamwork) or clinical risk management programs (such as medical education, risk mitigation, peer program and communication and resolution) for Question 2. Terms relating to ‘outcomes’ included malpractice, negligence, complaint, claim management and medico-legal.

We searched three bibliographic databases (PubMed, Scopus and Web of Science) and grey literature sources (Google, Proquest Theses, GreyLit.org and Mednar) for relevant studies. The reference lists and citation searching of included studies were included as other search methods. To ensure applicability to a modern healthcare system only studies published since 2011 were included. The search was conducted first in September 2022 and then repeated in July 2023.

Screening based on title and abstract was conducted independently in pairs by four members of the research team (TS, MZ, JG, JR) following training on two sets of 100 studies.

### Quality appraisal

The quality of included studies was appraised independently in pairs by four members of the research team (TS, JG, JR, PH) using AMSTAR 2 for systematic reviews [22] and National Institute of Health tools for case–control studies and uncontrolled pre-post studies [23]. These tools include 16 items (systematic reviews) or 12 items (case–control studies and uncontrolled pre-post), which were scored as ‘Yes’, ‘No’, ‘Not applicable’ or ‘Cannot determine’ [23], AMSTAR 2 also uses ‘Probably yes’.

### Data collection

Data was extracted from each paper into a Microsoft Excel spreadsheet that had been pilot tested by three reviewers. Extraction was conducted by a single reviewer (TS or MZ) and then checked by a second reviewer (JG, JR).

## Synthesis

A narrative synthesis was used to describe the key findings for both review questions. For review Question 1, results are presented separately for each patient characteristic, grouped according to patient demographics (e.g. age, sex, complainant), patient risk factors (e.g. American Society of Anaesthesiologists’ (ASA) score, the existence of a mental disorder, re-operation) and the therapeutic context (e.g. aspects of treatment, diagnosis, setting and/or phase of care including length of stay (LOS) and complications). For review Question 2, results are presented for seven different types of programs implemented to reduce the number of complaints and/or claims against doctors. The consistency, clinical impact, generalisability, and applicability of study findings were appraised using the NHRMC matrix which ranks each component’s strength using a four-point scale (excellent, good, satisfactory and poor) [20].

## Results

### Literature search

**Fig. 1:**
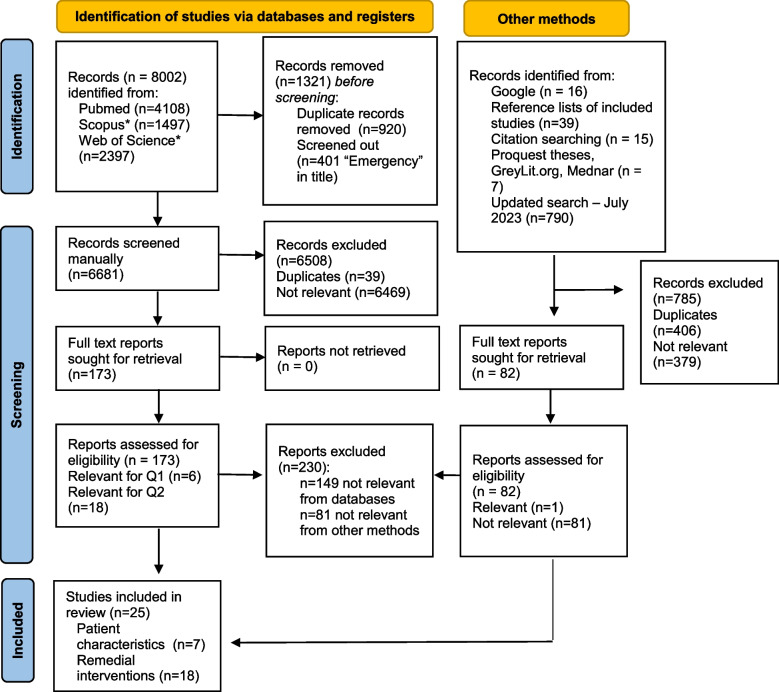
PRISMA study flow diagram [19]. * filters applied to these search results (Australia, New Zealand, Canada, UK)

Nearly 8900 studies were identified across the search strategy, of which 255 full texts were reviewed (Fig. 1). Of these, 230 were excluded as not relevant or due to an ineligible study design. A total of seven studies were included for Question 1, and 18 studies were included for Question 2 (Supplementary Table S4).

### Question 1

The characteristics of the studies included for Question 1 are presented in Table 2. There were six comparative studies with concurrent controls (three from the USA [24–26], two from the UK [27, 28]) and one from Italy [29] and one systematic reviews of non-randomised control trials [3]. The in-patient hospital setting was most common (*n* = 5) across a range of specialties and conditions, most commonly surgery. In total, there were 27 variables reported across the seven studies, 17 of these were included in multiple studies. Sex (*n* = 6) and age (*n* = 5) were the most frequently recorded patient demographics. For patient risk factors, ASA score, mental disorders, tobacco use and body mass index (BMI) > 30 were measured in two studies. For therapeutic context, LOS, setting, complications and treatment were measured in two studies.

**Table 2:** Characteristics of seven included studies for Question 1

First author (year) [citation]	Design	Critical appraisal			Country	Setting	Specialty	Condition	Type	Warranted or unwarranted	Patient characteristics
		Y	N	NA or CD							
Facchin (2023) [29]	A comparative study with concurrent controls	6	4	2	Italy	In-patient hospital	Bariatric surgery	Obesity	Malpractice claims	Both	Type of body contouring procedure
Grandizio (2021) [24]	A comparative study with concurrent controls	8	2	2	USA	Mixed	Hand surgery	Hand surgery	Complaint	n/s	Age, sex, BMI > 30, race, marital status, employment status, tobacco use, insurance status, mental behavioural or neurological disorder, diagnosis, treatment, complications
Jones (2021) [27]	A comparative study with concurrent controls	8	2	2	UK	In-patient hospital	Neurosurgery	Chronic subdural haematoma	Complaint	n/s	Age, sex, complainant, ASA score, referred from other hospital, LOS, time from admission to operation, reoperation, complications
Kynes (2013) [25]	A comparative study with concurrent controls	9	2	1	USA	In-patient hospital	Anaesthesiology	Mixed	Complaint	n/s	Age, sex, race, procedural features (e.g. use of anaesthesia, actual minus scheduled start time), ASA score
Rae (2022) [26]	A comparative study with concurrent controls	9	1	2	USA	In-patient hospital	Orthopaedic surgery	Spinal surgery	Complaint	n/s	Age, sex, BMI > 30, race, marital status, employment status, tobacco use, insurance status, treatment (surgery), mental behavioural or neurological disorder, worker's compensation
Reader (2014) [3]	Other: systematic review of non-RCTs or literature review	4	7	5	–	Mixed	Mixed	Mixed	Complaint	n/s	Sex, complainant, setting
Robin Taylor (2020) [28]	A comparative study with concurrent controls	10	0	2	UK	In-patient hospital	Medical and surgical wards	End of life	Complaint	n/s	Age, sex, expected death, setting, LOS, advance plans, clinical 'problems', non-beneficial interventions, harms, treatment escalation limitation plan (TELP)

*Acronyms*: *ASA* American Society of Anaesthesiologists, *BMI* Body Mass Index, *LOS* Length of stay, *UK* United Kingdom, *USA* United States of America, *Y* ‘Yes’, *N* ‘No’, *NA* ‘Not applicable’, *CD* ‘Cannot determine’, *n/s* not specified

Quality assessment is summarised in Table 2, Supplementary Table S5 (comparative studies) and Supplementary Table S6 (systematic reviews). For the 6 comparative studies, 6 to 10 (mean 8.3, SD = 1.4) of 12 criteria were met; for the systematic review, 4 of the 16 criteria were met (or probably met).

In general, there was very limited evidence for the existence of significant relationships between patient characteristics and the rate of complaints or claims (Table 3). For demographics, one study identified that a 10-year increase in the age of paediatric surgery patients led to a near 50% greater odds (OR = 1.47, CI 1.04–2.08) of a complaint and that male gender reduced odds of a complaint in adults by 34% (OR = 0.66, CI 0.47–0.92) [25]. However, sex and age were not significant predictors in five and four other studies, respectively. A systematic review of 36 studies (comprising 44,211 complaints) estimated that 64% of complainants were patients and 26% were family members; the remaining 10% was not specified [3]. Of patient risk factors, patients with mental, behavioural, or neurodevelopmental disorders were significantly more likely to complain following hand and upper extremity surgery [24] and spine surgery [26] (Table 3).

**Table 3:**
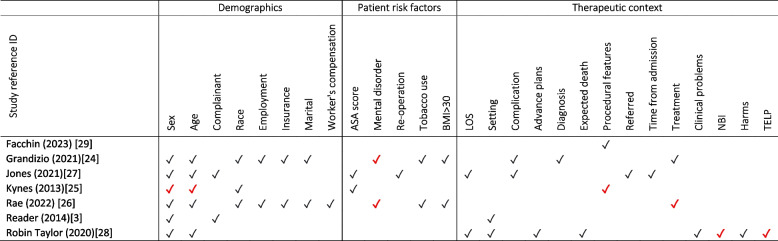
Summary of patient characteristics included (✓) in Question 1 studies

*ASA* American Society of Anaesthesiologists, *BMI* Body Mass Index, *LOS* Length of stay, *NBI* Non-beneficial interventions, *TELP* Treatment escalation limitation plan

A  indicates a significant relationship between the characteristic and the rate of complaints or claims

References: [3, 24–29]

In terms of therapeutic context, there were lower odds of a complaint for two procedural features: (i) use of a general anaesthetic in both paediatric and adult populations provided odds ratios, respectively, of 0.22 (CI 0.07–0.62) and 0.67 (CI 0.47–0.95) compared to no general anaesthetic, and (ii) a 1-h delay in actual start time led to slightly higher odds of a complaint, more notably in paediatrics (OR = 1.27, CI 1.10–1.47) than in adults (OR = 1.05, CI 0.95–1.16) [25]. The odds of a complaint were seven times greater for patients undergoing surgery (CI 5.2–9.6) [26]. The overuse of non-beneficial interventions and underuse of treatment escalation plans predicted complaints from the next-of-kin of patients who died in hospital [28]. For example, treatment escalation limitation plans were used significantly less frequently in complaints (23.8% versus 47.2%, *P* = 0.013) [28]. Other components of therapeutic context, including LOS, setting, and experiencing complications and harms, were not significant predictors of complaints (Table 3).

### Question 2

Uncontrolled pre-post studies (*n* = 14) were the most common study design included for Question 2, followed by comparative studies with concurrent controls (*n* = 2) and systematic reviews (*n* = 2) (Table 4). Studies were set in the USA (*n* = 12) [13, 15, 30–39], Canada (*n* = 2) [40, 41], the UK [12], Ireland [42] and New Zealand [43] (*n* = 1, each). The studies addressed malpractice claims (*n* = 9), complaints (*n* = 5), and regulatory notifications (*n* = 2) and a mix of outcomes (*n* = 1). In-patient hospital (*n* = 11) was the most common setting, followed by mixed (*n* = 4), primary care and secondary care (*n* = 1, each). There were seven types of interventions for Question 2 studies: risk management (*n* = 6), CRPs (*n* = 5) (note one study [31] assessed both), medical remediation (*n* = 3), peer program (*n* = 2) and, CPD, simulation training and shared decision-making (*n* = 1, each). Quality assessment is summarised in Table 3, Supplementary Table S5 (comparative studies), Table S7 (uncontrolled pre-post studies) and Supplementary Table S6 (systematic reviews). Eight of the 12 criteria were met for the one comparative study; 3 to 11 of the 12 criteria were met for the 14 uncontrolled pre-post studies (mean 7.6, SD = 2.6); and 8 and 11 of the 16 criteria were met for the two systematic reviews.

**Table 4:** Characteristics of 18 included studies for Question 2

First author (year) [citation]	Design NHMRC	Critical appraisal			Country	Setting	Specialty	Condition	Type	Warranted or unwarranted	Intervention type
		Y	N	NA or CD							
Adams (2014) [15]	Case series	8	2	2	USA	In-patient hospital	Gastroenterology	Gastrointestinal diseases	Malpractice claims	n/s	Communication and resolution program
Barragry (2016) [42]	Case series	9	1	2	Ireland	Primary care	General practice	Mixed	Complaint	n/s	Risk management program
Cardoso (2017) [31]	Other—Systematic review of non-RCTs	9	2	5	USA	Secondary care (specialist)	Obstetrics	Obstetrics and gynaecology	Malpractice claims	n/s	Communication and resolution program; Risk management program
Cosman (2011) [30]	Case series	4	3	4	USA	In-patient hospital	General surgery	n/a	Regulatory	n/s	Medical remediation program
Diraviam (2018) [32]	Case series	3	5	4	USA	In-patient hospital	Mixed	Mixed	Malpractice claims	n/s	Risk management program
Durand (2015) [44]	A systematic review of Level II studies	11	2	3	–	Mixed	Mixed	Mixed	Malpractice claims	n/s	Shared decision-making
Fustino (2019) [33]	Case series	6	3	3	USA	In-patient hospital	Mixed	Mixed	Complaint	n/s	Communication and resolution program
Juo (2019) [34]	Case series	6	5	1	USA	In-patient hospital	General surgery	n/a	Malpractice claims	n/s	Risk management program
Kachalia (2018) [13]	A comparative study with concurrent controls	8	3	1	USA	In-patient hospital	Mixed	Mixed	Malpractice litigation	n/s	Communication and resolution program
LeCraw (2018) [35]	Case series	10	1	1	USA	In-patient hospital	Mixed	Mixed	Malpractice claims	n/s	Communication and resolution program
Lillis (2014) [43]	Case series	7	4	1	New Zealand	Mixed	Mixed	n/a	Regulatory	n/s	Medical remediation program
Milne (2013) [40]	Case series	6	5	1	Canada	In-patient hospital	Mixed	Obstetrics and gynaecology	Malpractice claims	n/s	Risk management program
Nassiri (2019) [36]	Case series	11	1	0	USA	In-patient hospital	Otolaryngology	u/k	Complaint	n/s	Peer program
O'Brien (2014) [12]	Case series	8	4	0	UK	Mixed	Mixed	n/a	Mix	n/s	Medical remediation program
Pichert (2013) [25]	Case series	11	1	0	USA	In-patient hospital	Mixed	u/k	Complaint	n/s	Peer program
Raper (2017) [38]	Case series	7	2	3	USA	In-patient hospital	General surgery	Surgical	Malpractice claims	n/s	Risk management program
Schaffer (2021) [39]	Case series	11	0	1	USA	In-patient hospital	Obstetrics and gynaecology	Obstetrics and gynaecology	Malpractice claims	n/s	Simulation training
Wenghofer (2015) [41]	A comparative study with concurrent controls	8	3	1	Canada	Mixed	Mixed	n/a	Complaint	Warranted	Continuing professional development

*Acronyms*: *UK* United Kingdom, *USA* United States of America, *Y* ‘Yes’, *N* ‘No’, *NA* ‘Not applicable’, *CD* ‘Cannot determine’, *n/s* not specified

Findings and definitions for Question 2 across the seven types of interventions and eight included outcomes are presented in Table 5. No studies examined doctor satisfaction or patient outcomes (such as mortality or morbidity).

**Table 5:** Summary of findings from 17 studies included for Question 2 across eight outcomes and seven types of intervention

Type of intervention	Definition	Total	↓Claims	↓ Complaints	↓ Claims costs, or premiums	More timely management	↓ Doctor risk profile/ ↑ performance	↑ Staff confidence/knowledge	↑ Culture	↑ Patient satisfaction
Risk management program	“a formal approach encompassing evaluation of complaints, improved communication in relation to complaints, and more direct use of insights gained from complaints analysis” [42]	6	✓✓✓ [32, 38, 40] ~ [31]	✓ [42]	✓✓✓✓ [31, 32, 38, 40]	✓[42]	–	✓✓[34, 40]	✓[40]	–
Communication and resolution program	CRPs aim to better communicate adverse events to patients, investigate and explain what happened; provide emotional support; and apologise and proactively offer compensation if appropriate [35]. CRPs involve communication between doctor and patient outside the court setting to reach a mutual agreement to resolve the dispute and fair compensation and include apology laws in which apologies made by medical practitioners cannot be used as evidence in medical malpractice litigation [31]	5	✓✓✓ [15, 31, 35] ~ [13]	✓ [33]	✓✓✓ [15, 31, 35] ~ [13]	✓✓✓ [15, 31, 35] ~ [13]	–	–	–	✓ [33]
Medical remediation	The process by which a doctor’s poor performance is ‘remedied’, which permits the doctor to return to safe practice [45]. It is formally defined as ‘an intervention, or suite of interventions, required in response to assessment against threshold standards’, with thresholds set by regulatory bodies (e.g. AHPRA in Australia) to keep patients safe [46]	3	✓ [12]	–	–	–	✓✓ [30, 43]	–	–	–
Peer program	An organised effort whereby people (peers) critically appraise, systematically assess, monitor, make judgements, determine their strengths and weaknesses and review the quality of their practice, to provide evidence to use as the basis of recommendations by obtaining the opinion of their peers” [47, 48]. The use of peer messengers (doctors) involves the provision of feedback to doctors deemed at higher risk of experiencing a patient complaint or malpractice claim	2	–	✓ [36]	–	–	✓ [37]	–	–	–
Shared decision-making	“Involving a patient and health care provider who work together to deliberate about the harms and benefits of two or more reasonable options, in order to choose a course of care that is ideally aligned with the patient’s preferences” (p. 2) [44]	1	~ [44]	–	–	–	–	–	–	–
Simulation training	“A technique for practice and learning that can be applied to many different disciplines and types of trainees. It is a technique (not a technology) to replace and amplify real experiences with guided ones, often ‘immersive’ in nature, that evoke or replicate substantial aspects of the real world in a fully interactive fashion” (p. 349) [49]	1	✓ [39]	–	~ [39]	–	–	–	–	–
Continuing professional development	A range of activities undertaken to maintain clinical skills and knowledge, as well as competence in the delivery of patient-centred care [50]. Participation in CPD is mandatory for doctors in several countries, including Australia and Canada, while being used to evaluate maintenance of competence in the USA	1	–	✓ [41]	–	–	–	–	–	–

*CPD* Continuing professional development

↓ decrease, ↑ increase, ✓ a study reporting a better outcome (e.g. reduced claims rate), ~ a study reporting no evidence of effect

Each ✓ and ~ indicates a study, including the citation

The six studies of risk management programs [31, 32, 34, 38, 40, 42], also called risk reduction programs, were heterogeneous in nature, and included enhanced evaluation of, and response to, complaints [42], active engagement of physicians in risk assessment [32], lectures followed by a mock lawsuit [34], and education [38, 40]. Evidence from these studies of risk management programs supported reductions in claims, complaints and claims costs (Table 5). Other benefits included more timely complaints management, improved patient safety culture and staff confidence.

Evidence for communication and resolution programs (CRPs, five studies [13, 15, 31, 33, 35]) was consistent across four studies. There were lower rates of claims and complaints, lower claim amounts, and faster resolution of claims following the implementation of CRPs (Table 5) [15, 31, 33, 35]. However, results were less supportive in a study using an interrupted time series (ITS) design [13]. One study demonstrated improved patient satisfaction [33].

Three studies of medical remediation showed either a reduction in claims rates [12] or an improved doctor risk profile [29, 43].

Two studies of peer review, or the use of peer messengers, demonstrated a reduction in either complaint rates [36] or improved doctor risk profile [37] (Table 5).

A systematic review of five studies concluded that there was insufficient evidence to determine whether or not shared decision-making reduces claims [44]. A retrospective pre-post program evaluation of simulation training on malpractice claims among obstetrician-gynaecologists reported that the rate of claims after simulation training was halved to 5.7 claims per 100 physician years of coverage. Attending more sessions was associated with a greater reduction in claims, although there was no difference in the total costs of paid claims before and after the training [39].

In one included study of CPD, doctors who reported participation in CPD activities were significantly less likely (OR 0.60; CI 0.39 to 0.95) to receive quality of care-related complaints than those who did not report participating in CPD [41]. Participants in group-based CPD were less likely (OR 0.68; CI 0.47 to 0.98) to receive quality of care-related complaints than individual or assessment-based CPD [41].

### Summary of the evidence

A summary of the included studies’ evidence base, consistency, clinical impact, generalisability and applicability is included in Table 6. The evidence base was rated as poor for both Question 1 and 2 (Table 6). Consistency and clinical impact were slightly higher for Question 2 than Question 1, whereas generalisability and applicability were satisfactory for both Question 1 and Question 2.

**Table 6:** NHMRC matrix summary for Question 1 and Question 2

Component	A	B	C	D
	Excellent	Good	Satisfactory	Poor
Evidence base				Q1 Q2
Consistency		Q2^a^	Q1^a^	
Clinical impact			Q2	Q1
Generalisability			Q1 Q2	
Applicability			Q1 Q2	

*Q1* Question 1, *Q2* Question 2

^a^Consistency based on narrative synthesis rather than meta-analysis and *I*^2^

The evidence base is assessed in terms of the quantity, level and quality (risk of bias) of the included studies

Consistency assesses whether the findings are consistent across the included studies (including across a range of study populations and study designs)

Clinical impact is a measure of the potential benefit from the application of the guideline to a population

Generalisability assesses whether the subjects and settings of the included studies match the patient population being targeted and the clinical setting where the recommendation will be implemented

Applicability addresses whether the evidence base is relevant to the Australian health care system generally

## Discussion

This review has identified a clear lack of recent high-quality studies to inform an in-depth understanding of either review Question 1 or Question 2. For Question 1, seven patient characteristics were associated with patients’ likelihood to complain or make a medico-legal claim against a doctor; however, only one of these findings (presence of a mental disorder) was replicated. This may be related to the paucity of studies, for example, only half of the patient characteristics were evaluated in more than one study. While more studies were included for Question 2, the low quality of the predominant study design (case series) severely limits the strength of the review’s findings.

The main finding for Question 1 of a relationship between a patient’s mental health status and complaint behaviour may reflect non-modifiable associations between underlying mental health conditions, poorer outcomes and reduced satisfaction after surgery [24, 26]. Alternatively, the finding may reflect the impact of stigma experienced by these patients in healthcare settings. Mental illness-related stigma is prevalent in healthcare [51]. Stigma creates barriers to accessing healthcare, such as delays in help-seeking, treatment discontinuation, suboptimal therapeutic relationships, patient safety concerns and poorer quality care [52]. The presence of these barriers may be associated with a complaint about a healthcare provider.

Findings for Question 2 offer some evidence to support most of the included interventions, particularly risk management programs and CRPs. Some of the commonly occurring attributes of risk management programs were the evaluation and analysis of complaints and claims, targeted medico-legal education, and implementation of patient safety measures. The majority of the risk management programs were developed and delivered internally, either at the level of hospital department [38], hospital-wide [32, 34] or general practice-level [42]. Local contextualisation, incorporating the site-specific nature of malpractice claims and legislation, and delivery of risk management programs apparently enhance the acceptability of risk management programs for surgeons, in particular [53–55]. Nevertheless, in one study, the Society of Obstetricians and Gynaecologists of Canada partnered with a healthcare insurance representative body to support the international expansion of a risk management program [40].

Studies of CRPs were generally consistent in showing lower rates of claims and complaints, lower claim amounts, and faster resolution of claims following the implementation of CRPs. However, limited adherence to the key components of CRP, including a proliferation of partial apology laws, may detract from the effectiveness of CRP in meeting the needs of injured patients [56–58]. Patients involved in CRP have expressed a greater desire for information provision from hospitals about efforts to prevent recurrences of the event [59].

Interventions such as caps on compensation, attorney fees, and alternative payment systems and liabilities [31] were excluded from the review as they are not doctor-directed interventions. The impacts of these medical malpractice reforms have been recently summarised [60, 61].

The small number of included studies (Question 1) and the low quality of included studies (Question 2) represent major gaps in the evidence. For Question 1, there were a large number of excluded studies that were uncontrolled or unadjusted cross-sectional studies of complaints or claims that simply report the underlying characteristics of a claims database. Due to the lack of a control group, these studies do not provide particularly useful insights into the relationship between patient characteristics and the rate of complaints or claims. While more studies were included for Question 2, the predominant study design (i.e. uncontrolled pre-post) is weak as it does not permit adjustments for other secular trends in claims or confounders, or include control sites. Therefore, very little strength could be offered for recommendations emanating from either Question 1 or Question 2.

For Question 1, only one study specified whether a complaint was warranted or unwarranted [41]. No study included both types of complaints to determine predictors of successful interventions targeting unwarranted claims/complaints. The finding that a substantial subset of complaints originate from non-patient sources is likely to reduce the predictive value of patient characteristics for claims and complaints in this analysis. For Question 2, no studies assessed staff satisfaction or patient outcomes, such as mortality or morbidity. Additionally, there is rarely any evidence provided about generalisability or the potential for implementation and sustainability of the intervention, and most studies are limited to a single hospital/health service. Only one included study reported on the impact on organisational culture [40] or patient satisfaction [33].

All stages of the rapid review were conducted independently in duplicate to minimise the risk of errors. However, we only included studies published since 2011. This may have excluded relevant, older literature, which may be a limitation to this rapid review. Additionally, we filtered search results from the Scopus and Web of Science databases to countries with similar health systems (Australia, New Zealand, Canada and the UK) and screened out studies with ‘emergency’ in the title.

## Conclusions

Despite substantial efforts made to collect information about patient complaints and claims, research has generally failed to robustly determine patient characteristics associated with complaints and claims. There is a small amount of evidence that patients with mental health conditions are more likely to complain.

The evidence for the effectiveness of interventions to reduce the likelihood of a doctor receiving a complaint or claim is also weak, as it is dominated by low-quality, uncontrolled pre-post studies. Only one or two studies were included for five types of programs (peer programs, medical remediation, shared decision-making, simulation training and CPD). More evidence, however, offers support for the effectiveness of risk management programs and CRPs in reducing complaints and claims.

### Supplementary Information

**Additional file 1: Table S1.** Pubmed search - 8 September 2022. **Table S2.** Scopus search - 8 September 2022. **Table S3.** Web of Science - 8 September 2022. **Table S4.** Summary of study design for included studies for Question 1 and 2 using NHMRC levels of evidence [20]. **Table S5.** Summary of quality appraisal for eight comparative studies with concurrent controls, six for Question 1 (Q1) and two for Question 2 (Q2). **Table S6.** Summary of quality appraisal for three systematic reviews (one for Question 1 (Q1) and two for Question 2 (Q2)). **Table S7.** Summary of quality appraisal for 14 uncontrolled pre-post studies for Question 2 (Q2).

## Data Availability

The datasets analysed during the current study are available from the corresponding author on reasonable request.
