# Supplementary material for: Patient characteristics of, and remedial interventions for, complaints and medico-legal claims against doctors: a rapid review of the literature
Source: Syst Rev. 2024 Apr 9;13:104. doi: 10.1186/s13643-024-02501-8 (PMC11003134; doi:10.1186/s13643-024-02501-8)
Supplement: Supplementary file 1 — Additional file 1: Table S1. Pubmed search - 8 September 2022. Table S2. Scopus search - 8 September 2022. Table S3. Web of Science - 8 September 2022. Table S4. Summary of study design for included studies for Question 1 and 2 using NHMRC levels of evidence [20]. Table S5. Summary of quality appraisal for eight comparative studies with concurrent controls, six for Question 1 (Q1) and two for Question 2 (Q2). Table S6. Summary of quality appraisal for three systematic reviews (one for Question 1 (Q1) and two for Question 2 (Q2)). Table S7. Summary of quality appraisal for 14 uncontrolled pre-post studies for Question 2 (Q2). [file 13643_2024_2501_MOESM1_ESM.docx]

Supplementary Tables

Table S-1 Pubmed search - 8 September 2022

| Search number | Query | Filters | Results |
| --- | --- | --- | --- |
| 1 | "medical officer*"[Title/Abstract] OR "doctor*"[Title/Abstract] OR "physician*"[Title/Abstract] OR "medicine"[MeSH Terms] OR "health service"[Title/Abstract] |  | 1,744,081 |
| 2 | "malpractice"[Title/Abstract] OR "negligen*"[Title/Abstract] OR "patient complaint*"[Title/Abstract] OR "closed claim*"[Title/Abstract] OR "open claim*"[Title/Abstract] OR "claim manage*"[Title/Abstract] OR "malpractice"[MeSH Terms] OR "insurance, liability"[MeSH Terms] OR "professional misconduct"[MeSH Terms] OR "medical defence*"[Title/Abstract] OR "medical regulat*"[Title/Abstract] OR "medicolegal*"[Title/Abstract] OR "medico legal*"[Title/Abstract] OR "medical errors"[MeSH Terms] |  | 178,858 |
| 3 | "patient characteristic*"[Title/Abstract] OR "demograph*"[Title/Abstract] OR "sociodemograph*"[Title/Abstract] OR "medical histor*"[Title/Abstract] OR "socio economic*"[Title/Abstract] OR "SES"[Title/Abstract] OR "patient factor*"[Title/Abstract] OR "risk factor*"[Title/Abstract] OR "patient attribute*"[Title/Abstract] OR "health literacy"[Title/Abstract] OR "health literacy"[MeSH Terms] |  | 1,277,476 |
| 4 | "evidence based care bundle"[Title/Abstract] OR "patient care bundles"[MeSH Terms] OR "simulation training"[Title/Abstract] OR "simulation training"[MeSH Terms] OR "patient safety"[Title/Abstract] OR "patient safety"[MeSH Terms] OR "safety checklist*"[Title/Abstract] OR "standardisation"[Title/Abstract] OR "standardization"[Title/Abstract] OR "communication"[Title/Abstract] OR "health communication"[MeSH Terms] OR "teamwork"[Title/Abstract] OR "crew resource management, healthcare"[MeSH Terms] OR "handover"[Title/Abstract] OR "clinical handover"[Title/Abstract] OR "patient handoff"[MeSH Terms] |  | 415,299 |
| 5 | "Medical education"[Title/Abstract] OR "education, medical, continuing"[MeSH Terms] OR "risk mitigation"[Title/Abstract] OR "risk management"[Title/Abstract] OR "risk management"[MeSH Terms] OR "empathy training"[Title/Abstract] OR "empathy"[MeSH Terms] OR "Informed consent training"[Title/Abstract] OR "informed consent"[MeSH Terms] OR "open disclosure"[Title/Abstract] OR "peer program"[Title/Abstract] OR "communication and resolution"[Title/Abstract] OR "continuing professional development"[Title/Abstract]) OR "remediation"[Title/Abstract] OR "remedia*"[Title/Abstract] OR "reskilling"[Title/Abstract] OR "re-skilling"[Title/Abstract] OR "retraining"[Title/Abstract] OR "re-training"[Title/Abstract] OR "simulation"[Title/Abstract] |  | 486,923 |
| 6 | #4 or #5 |  | 873,547 |
| 7 | #3 or #6 |  | 2,067,502 |
| 8 | #1 and #2 and #7 |  | 9,319 |
| 9 | #1 and #2 and #7 | English | 8,454 |
| 10 | #1 and #2 and #7 | English, from 2011 - 2023 | 4,108 |

Table S-2 Scopus search - 8 September 2022

| Search number | Query | Filters | Results |
| --- | --- | --- | --- |
| 1 | "medical officer*" OR "doctor*" OR "physician*" OR "health service" |  | 1,771,700 |
| 2 | malpractice OR "negligen*" OR "patient complaint*" OR "closed claim*" OR "open claim*" OR "claim manage*" OR "malpractice" OR "medical defence*" OR "medical regulat*" OR "medicolegal*" OR "medico legal*" OR "medical errors" OR "professional misconduct" |  | 135,232 |
| 3 | "patient characteristic*" OR "demograph*" OR "sociodemograph*" OR "medical histor*" OR "socio economic*" OR "SES" OR "patient factor*" OR "risk factor*" OR "patient attribute*" OR "health literacy" |  | 3,053,434 |
| 4 | "evidence based care bundle" OR "patient care bundles" OR "simulation training" OR "patient safety"OR "safety checklist*"OR "standardisation" OR "standardization" OR "communication" OR "teamwork" OR "crew resource management" OR "handover" OR "patient handoff" |  | 2,683,446 |
| 5 | "Medical education" OR "risk mitigation" OR "risk management" OR "empathy training" OR "informed consent" OR "open disclosure" OR "peer program" OR "communication and resolution" OR "continuing professional development" OR "remediation" OR "remedia*" OR "reskilling" OR "re-skilling" OR "retraining" OR "re-training" OR "simulation" |  | 558,386 |
| 6 | #4 or #5 |  | 3,176,929 |
| 7 | #3 or #6 |  | 6,095,114 |
| 8 | #1 and #2 and #7 |  | 14,994 |
| 9 | #1 and #2 and #7 | limit English | 13,621 |
| 10 | #1 and #2 and #7 | limit English, >2010 | 5879 |
|  |  | limit AU, NZ, CA, UK | 1497 |

Table S-3 Web of Science - 8 September 2022

| Search number | Query | Filters | Results |
| --- | --- | --- | --- |
| 1 | medical officer* OR doctor* OR physician* OR health service |  | 923,305 |
| 2 | malpractice OR negligen* OR patient complaint* OR closed claim* OR open claim* OR claim manage* OR malpractice OR medical defence* OR medical regulat* OR medicolegal* OR medico legal* OR medical errors OR professional misconduct |  | 209,988 |
| 3 | patient characteristic* OR demograph* OR sociodemograph* OR medical histor* OR socio economic* OR SES OR patient factor* OR risk factor* OR patient attribute* OR health literacy |  | 3,341,468 |
| 4 | evidence based care bundle OR patient care bundle* OR simulation training OR patient safety OR safety checklist* OR standardisation OR standardization OR communication OR teamwork OR crew resource management OR handover OR patient handoff |  | 1,852,131 |
| 5 | Medical education OR risk mitigation OR risk management OR empathy training OR informed consent OR open disclosure OR peer program OR communication and resolution OR continuing professional development OR remediation OR remedia* OR reskilling OR re-skilling OR retraining OR re-training OR simulation |  | 775,393 |
| 6 | #4 or #5 |  | 2,549,833 |
| 7 | #3 or #6 |  | 5,457,457 |
| 8 | #1 and #2 and #7 |  | 18,061 |
| 9 | #1 and #2 and #7 | limit English | 16,582 |
| 10 | #1 and #2 and #7 | limit English, >2010 | 11,431 |
|  |  | limit AU, NZ, CA, UK | 2397 |

Table S-4 Summary of study design for included studies for Question 1 and 2 using NHMRC levels of evidence [20].

| Level | Study design | Question 1 | Question 2 | Total |
| --- | --- | --- | --- | --- |
| I | A systematic review | 1 | 2 | 3 |
| III-2 | A comparative study with concurrent controls (i.e. non-randomised experimental trials, cohort studies, case-control studies, interrupted time series studies with a control group) | 6 | 2 | 8 |
| IV | Case series with either post-test or pre-test/post-test outcomes | 0 | 14 | 14 |
|  | Total included studies | 7 | 18 | 25 |

S-5 Summary of quality appraisal for eight comparative studies with concurrent controls, six for Question 1 (Q1) and two for Question 2 (Q2)

|  | Q1 |  |  |  |  |  |  | Q2 |  |
| --- | --- | --- | --- | --- | --- | --- | --- | --- | --- |
| Study design – Comparative study with concurrent control | Facchin (2023) [29] | Grandizio (2021) [24] | Jones (2021) [27] | Kynes (2013) [25] | Rae (2022) [26] | Robin Taylor (2020) [28] |  | Wenghofer et al 2015 [41] | Kachalia (2018)[13] |
| 1. Was the research question or objective in this paper clearly stated and appropriate? | N | Y | Y | Y | Y | Y |  | Y | Y |
| 2. Was the study population clearly specified and defined? | N | Y | Y | Y | Y | Y |  | N | Y |
| 3. Did the authors include a sample size justification? | NA | NA | NA | NA | NA | NA |  | N | N |
| 4. Were controls selected or recruited from the same or similar population that gave rise to the cases (including the same timeframe)? | Y | Y | Y | Y | Y | Y |  | Y | Y |
| 5. Were the definitions, inclusion and exclusion criteria, algorithms or processes used to identify or select cases and controls valid, reliable, and implemented consistently across all study participants? | Y | Y | Y | Y | Y | Y |  | Y | Y |
| 6. Were the cases clearly defined and differentiated from controls? | Y | Y | Y | Y | Y | Y |  | Y | N |
| 7. If less than 100 percent of eligible cases and/or controls were selected for the study, were the cases and/or controls randomly selected from those eligible? | NA | NA | NA | N | NA | CD |  | NA | NA |
| 8. Was there use of concurrent controls? | Y | Y | Y | Y | Y | Y |  | Y | Y |
| 9. Were the investigators able to confirm that the exposure/risk occurred prior to the development of the condition or event that defined a participant as a case? | Y | Y | Y | Y | Y | Y |  | Y | Y |
| 10. Were the measures of exposure/risk clearly defined, valid, reliable, and implemented consistently (including the same time period) across all study participants? | Y | Y | Y | Y | Y | Y |  | Y | Y |
| 11. Were the assessors of exposure/risk blinded to the case or control status of participants? | N | N | N | N | N | Y |  | N | N |
| 12. Were key potential confounding variables measured and adjusted statistically in the analyses? If matching was used, did the investigators account for matching during study analysis? | N | N | N | Y | Y | Y |  | Y | Y |

Y - ‘Yes’, N - ‘No’, NA - ‘Not applicable’, CD -‘Cannot determine’; Q1 – Question 1, Q2 – Question 2

S-6 Summary of quality appraisal for three systematic reviews (one for Question 1 (Q1) and two for Question 2 (Q2))

|  | Q1 |  | Q2 |  |
| --- | --- | --- | --- | --- |
| Study design – Systematic review | Reader et al 2014 [3] |  | Cardoso (2017)[31] | Durand (2015)[44] |
| 1. Did the research questions and inclusion criteria for the review include the components of PICO? | NA |  | Y | N |
| 2. Did the report of the review contain an explicit statement that the review methods were established prior to the conduct of the review and did the report justify any significant deviations from the protocol? | N |  | Y | Y |
| 3. Did the review authors explain their selection of the study designs for inclusion in the review? | N |  | Y | y |
| 4. Did the review authors use a comprehensive literature search strategy? | PY |  | Y | Y |
| 5. Did the review authors perform study selection in duplicate? | N |  | y | Y |
| 6. Did the review authors perform data extraction in duplicate? | Y |  | Y | y |
| 7. Did the review authors provide a list of excluded studies and justify the exclusions? | N |  | N | Y |
| 8. Did the review authors describe the included studies in adequate detail? | PY |  | PY | Y |
| 9. Did the review authors use a satisfactory technique for assessing the risk of bias (RoB) in individual studies that were included in the review? | N |  | NA | Y |
| 10. Did the review authors report on the sources of funding for the studies included in the review? | N |  | N | N |
| 11. If meta-analysis was performed did the review authors use appropriate methods for statistical combination of results? | NA |  | NA | NA |
| 12. If meta-analysis was performed, did the review authors assess the potential impact of RoB in individual studies on the results of the meta-analysis or other evidence synthesis? | NA |  | NA | NA |
| 13. Did the review authors account for RoB in individual studies when interpreting/discussing the results of the review? | N |  | NA | Y |
| 14. Did the review authors provide a satisfactory explanation for, and discussion of, any heterogeneity observed in the results of the review? | NA |  | Y | Y |
| 15. If they performed quantitative synthesis did the review authors carry out an adequate investigation of publication bias (small study bias) and discuss its likely impact on the results of the review? | NA |  | NA | NA |
| 16. Did the review authors report any potential sources of conflict of interest, including any funding they received for conducting the review? | Y |  | Y | Y |

Y - ‘Yes’, PY – ‘Probably yes’, N - ‘No’, NA - ‘Not applicable’; Q1 – Question 1, Q2 – Question 2

S-7 Summary of quality appraisal for 14 uncontrolled pre-post studies for Question 2 (Q2)

|  | Q2 |  |  |  |  |  |  |  |  |  |  |  |  |  |
| --- | --- | --- | --- | --- | --- | --- | --- | --- | --- | --- | --- | --- | --- | --- |
| Study design - Uncontrolled pre-post | Adams (2014)[15] | Barragry (2016)[42] | Cosman (2011) [30] | Diraviam (2018)[32] | Fustino (2019)[33] | Juo (2019)[34] | LeCraw (2018)[35] | Lillis (2014)[43] | Milne (2013)[40] | Nassiri (2019)[36] | O'Brien (2014)[12] | Pichert (2013)[25] | Raper (2017)[38] | Schaffer (2021)[39] |
| 1. Was the study question or objective clearly stated? | Y | Y | N | N | Y | N | Y | Y | N | Y | Y | Y | Y | Y |
| 2. Were eligibility/selection criteria for the study population prespecified and clearly described? | N | Y | Y | N | N | N | Y | Y | N | Y | Y | Y | N | Y |
| 3. Were the participants in the study representative of those who would be eligible for the test/service/intervention in the general or clinical population of interest? | Y | Y | Y | Y | Y | Y | Y | Y | Y | Y | Y | Y | CD | Y |
| 4. Were all eligible participants that met the prespecified entry criteria enrolled? | Y | Y | CD | CD | CD | CD | Y | Y | CD | Y | N | Y | CD | Y |
| 5. Was the sample size sufficiently large to provide confidence in the findings? | Y | Y | N | CD | Y | Y | Y | N | Y | Y | Y | Y | Y | Y |
| 6. Was the test/service/intervention clearly described and delivered consistently across the study population? | Y | Y | Y | N | Y | Y | Y | Y | Y | Y | Y | Y | Y | Y |
| 7. Were the outcome measures prespecified, clearly defined, valid, reliable, and assessed consistently across all study participants? | Y | Y | Y | N | Y | N | Y | Y | Y | Y | Y | Y | Y | Y |
| 8. Were the people assessing the outcomes blinded to the participants' exposures/interventions? | CD | CD | N | CD | CD | N | Y | N | N | N | N | N | N | CD |
| 9. Was the loss to follow-up after baseline 20% or less? Were those lost to follow-up accounted for in the analysis? | NA | NA | NA | NA | NA | Y | NA | Y | Y | Y | N | Y | NA | Y |
| 10. Did the statistical methods examine changes in outcome measures from before to after the intervention? Were statistical tests done that provided p values for the pre-to-post changes? | Y | Y | NA | Y | N | Y | Y | N | N | Y | Y | Y | Y | Y |
| 11. Were outcome measures of interest taken multiple times before the intervention and multiple times after the intervention (i.e., did they use an interrupted time-series design)? | N | N | N | Y | Y | N | N | N | Y | Y | N | Y | Y | Y |
| 12. If the intervention was conducted at a group level (e.g., a whole hospital, a community, etc.) did the statistical analysis take into account the use of individual-level data to determine effects at the group level? | Y | Y | NA | N | N | Y | Y | NA | N | Y | Y | Y | Y | Y |

Y - ‘Yes’, N - ‘No’, NA - ‘Not applicable’, CD -‘Cannot determine’; Q2 – Question 2
